# Traumatic Emphysema

**Published:** 1854-01

**Authors:** Walter Henry

**Affiliations:** Inspector General of Military Hospitals, &c.; Montreal


From the "Medical Chronicle".

*Traumatic Emphysema.* By WALTER HENRY, M. D., Inspector General of Military Hospitals, &c.

I have read with interest a good case of traumatic emphysema from broken ribs, well treated and well described, in the November number of the Chronicle. Having had a good deal of experience in such cases, and also in more serious ones, where a ball or a pointed weapon wounds the lungs, I send you a few brief remarks on the subject, hoping that a communication from an old medical campaigner in the Peninsula may not be unacceptable.

Fracture of the ribs, with emphysema, is generally, and where great violence has not been the cause, more formidable in appearance than reality. To an inexperienced surgeon, the sight of a main in great pain and distress, breathing with difficulty and with his chest inflated like a barrel, is very alarming. Every bystander, of course, considers the case quite hopeless; yet, in the majority of such instances, there is no great danger. Under judicious treatment, adapted to the opposite conditions of collapse and reaction, restricting the action of the external respiratory muscles, and making the breathing as much phrenic as possible, with the employment of suitable medicines, nature soon effects a cure.

The first thing usually done after recovery from the immediate shock, is to apply a bandage round the chest; and, under ordinary circumstances, and when there is no hæmorrhage from the mouth, this is, doubtless, quite right. But it should be borne in mind, that a common calico or linen bandage, however scientifically applied, will soon slacken, cease to confine the chest effectually, and become nearly useless for the chief purpose of its employment. As soon, therefore, as the patient is removed to his own residence, or an hospital, a hempen or coarse linen vest, doubled, should be prepared and put on, which must be tightly fitted, and sewed, or laced down the back, and have straps to support it over the shoulders. This appears to me to be almost indispensable to the proper treatment of the case.

It is true that in some instances this constriction cannot be borne, being incompatible with respiration from the first; and in others it must be relaxed when inflammatory symptoms set in, even at the expense of disturbing the process of reunion in the broken ribs: still it is the most effectual treatment, but may be modified according to the state of the case, and the requirements of urgent symptoms.

When air is extensively diffused through the sub-cutaneous cellular tissues, and the patient is elderly or cachectic, the capillary circulation is so much impeded by its pressure, that the skin becomes cold, and of a livid, or nearly livid color, threatening gangrene. This is a most dangerous symptom, and the worst result is to be apprehended. I have only seen two instances of this bad description, both of which ended fatally, though stimulating liniments, gentle local friction, warm flannels, and appropriate constitutional treatment were sedulously employed.

In cases where there is no danger of this kind, friction and liniments are also useful in hastening the absorption of the extravasated air. Emphysema occassionally disappears rapidly, and I have seen a chest nearly one-fourth larger than usual restored to its normal dimensions in a single night. The precise *modus operandi* of this absorption is as mysterious as marvellous. The air cannot be supposed to re-enter the wounded bronchi, which have contracted, and a healing process is going on in them. The lymphatics, it must be supposed, are inadequate to absorption on this scale. It is difficult to believe that the veins are the agents, because we know, that air in any great quantity in these vessels destroys life. Cases have occurred where the internal jugular vein has been wounded during the excision of a deep-seated cervical tumor, in which the suction of a gulp of air has been audible, and instantly fatal. When emphysema suddenly disappears, many cubic inches of air must have been absorbed in a few hours, and I believe that physiology has not yet settled how this is accomplished, nor some other difficulties of the process of absorption generally.

I have never seen emphysema of the chest unaccompained by costal fracture or a wound; but we know from the authorities that this sometimes happens. Yet in some of the recorded cases, including the squeeze from an elephant, quoted in the article referred to, there is reason to suspect that undetected costal fracture may have taken place. Under certain circumstances of extensive emphysema, the crepitus of a broken rib may be very indistinct amidst the crepitus of the integuments. Limited, and what we may call idiopathic, emphysema has been caused occasionally by the violent efforts of puerperal women in restraining their breath.

This extravasation is not a very unusual accompaniment of sword and bayonet thrusts, and also, but more seldom, of bullet wounds. In the course of the Peninsular War, and the Nepaulese War in India, I have seen and treated many such cases, of which reports were forwarded to the proper authorities at the time. After a lapse of 35 years, or thereabouts, it is impossible to recollect them with distinctness; but my impression is that the presence of emphysema was considered a subordinate matter, and little regarded. It was even supposed, that when air thus found its way to the surface, there was less risk of hæmorrhage and internal inflammation.

In these cases, friction and stimulating unctuous applications were employed, and when the emphysema was extensive, and the skin tense, scarifications were attended with much benefit.

Of one case I retain a vivid recollection, because the patient was a friend and brother officer. He was wounded in one of the actions in the Pyrenees in July, 1813, by a musket ball, which passed deeply into the right lung and there remained. There was profuse hæmorrhage from the mouth immediately, with dreadful dyspnœa, and extensive emphysema over the upper part of the body. For the first three days he was in a most dangerous state, half suffocated, and only kept alive by frequent bleedings. During this time he lost more than two hundred ounces of blood, besides of the hæmorrhage.

My patient is now a General officer, residing in the south of England, in good health. He still carries the ball in his chest, where it has become encysted, nature having made a nest for it and the bits of cloth it carried in. It appears to rest upon the diaphragm, and the only inconvenience experienced from its presence is when sudden or violent bodily exertion is made; then alarming phrenic spasms occur. On this account the General, who was always fond of riding, although he still mounts his horse, cannot canter nor trot with any comfort.

A case of emphysema from broken ribs, very remarkable on account of the attending circumstances, occurred in Quebec in 1836. A soldier of the 66th Regiment, named Ramsay, was sitting on the outer edge of the rampart of the Citadel, gazing at the first spring ships coming round Point Levy. The place where he sat was immediately above the precipice, rising from the Lower Town, where there was no ditch nor glacis. By some carelessness he lost his balance and fell, first to the foot of the rampart, thirty-five feet, and then tumbled from rock to rock three hundred feet more, until he alighted on the roof of a house in Champlain Street.

I was then Surgeon of the 66th, and hastened to see the poor fellow. He was alive, but pulseless and insensible, and apparently dying. There were many bad cuts and lacerations over the body, from which the blood was still issuing; the chest and back were swollen and emphysematous, through which I could perceive the crepitus of broken ribs, though I could discover no fracture of the skull, nor other bones. After an hour's labor in restoring consciousness he at length recovered his senses, and was able to swallow a little brandy and water. I then proceeded to make a more careful examination of his body, and was pulling up his shirt for this purpose, when, after two or three deep sighs, he found utterance. It is natural to suppose that an expression of gratitude for his wonderful escape would have been his first exclamation, but poor Ramsay had other thoughts. His first words were, "*Ah, Doctor dear, dinna tear my sark!*"

This man had three of his right ribs broken, besides a large number of minor injuries. No doubt the numerous rocks projecting from the precipice broke his fall, and probably saved his life. His clothes were nearly cut to pieces, and each of these rents and tears was, no doubt, a quantity, however small, in the sum of resistance to the force of gravity. Besides, it is probable that the ribs were broken by the first fall to the foot of the rampart, when emphysema would take place. He was thus furnished with an elastic integument round the upper part of the body, which would, to a certain extent, defend him in his perilous descent, besides diminishing his specific gravity.

Ramsay was carefully removed to the Regimental Hospital, where his case excited much interest. In the evening there was great reaction, with high pyrexia and dyspnœa, such as might be expected under the circumstances, and large bleeding was required to subdue the inflammation. But after passing through a dangerous week, he finally recovered without permanent weakness or injury in the chest, and was discharged from hospital in about a month.

Montreal, Nov. 7, 1853.

